# Antibody response to different COVID-19 vaccines among the migrant workers of Bangladesh

**DOI:** 10.3389/fimmu.2023.1128330

**Published:** 2023-03-09

**Authors:** Md. Imam Hossain, Protim Sarker, Rubhana Raqib, Md Ziaur Rahman, Rezaul Hasan, Chloe K. Svezia, Mahbubur Rahman, Nuhu Amin

**Affiliations:** ^1^ Infectious Diseases Division, International Centre for Diarrhoeal Disease Research, Dhaka, Bangladesh; ^2^ Rollins School of Public Health, Emory University, Atlanta, GA, United States; ^3^ Institute for Sustainable Futures, University of Technology Sydney, Ultimo, NSW, Australia

**Keywords:** SARS-CoV-2, seroconversion, anti-spike-antibody, mRNA vaccines, booster dose, Bangladeshi migrant workers

## Abstract

**Background:**

Due to the ongoing COVID-19 pandemic, various host countries such as Singapore, imposed entry requirements for migrant workers including pre-departure COVID-19 seroconversion proof. To combat COVID-19 worldwide, several vaccines have acquired conditional approval. This study sought to assess antibody levels after immunization with different COVID-19 vaccines among the migrant workers of Bangladesh.

**Methods:**

Venous blood samples were collected from migrant workers who were vaccinated with different COVID-19 vaccines (n=675). Antibodies to SARS-CoV-2 spike protein (S) and nucleocapsid protein (N) were determined using Roche Elecsys^®^ Anti-SARS-CoV-2 S and N immunoassay, respectively.

**Results:**

All participants receiving COVID-19 vaccines showed antibodies to S-protein, while 91.36% were positive for N-specific antibodies. The highest anti-S antibody titers were found among the workers who completed booster doses (13327 U/mL), received mRNA vaccines Moderna/Spikevax (9459 U/mL) or Pfizer-BioNTech/Comirnaty (9181 U/mL), and reported SARS-CoV-2 infection in the last six months (8849 U/mL). The median anti-S antibody titers in the first month since the last vaccination was 8184 U/mL, which declined to 5094 U/mL at the end of six months. A strong correlation of anti-S antibodies was found with past SARS-CoV-2 infection (p < 0.001) and the type of vaccines received (p <0.001) in the workers.

**Conclusion**

Bangladeshi migrant workers receiving booster doses of vaccine, vaccinated with mRNA vaccines, and having past SARS-CoV-2 infection, mounted higher antibody responses. However, antibody levels waned with time. These findings suggest a need for further booster doses, preferably with mRNA vaccines for migrant workers before reaching host countries.

## Graphical Abstract

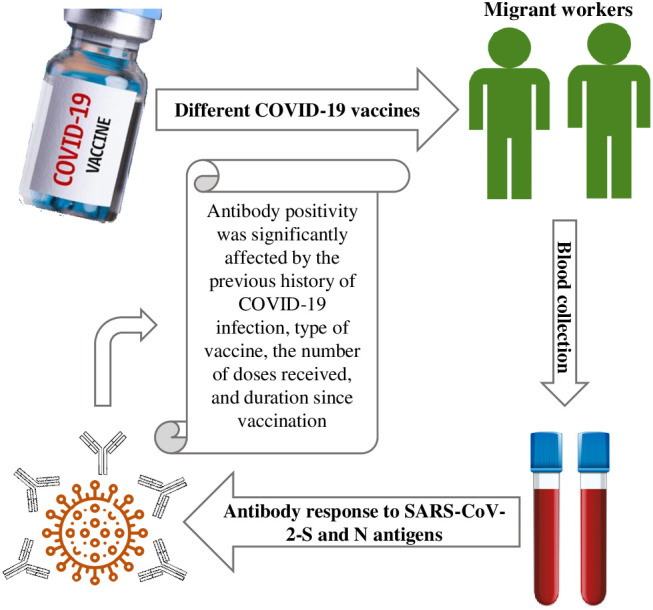

## Introduction

The first instance of COVID-19, a class of acute atypical respiratory illnesses in humans, was discovered in Wuhan, China, in December 2019 (1). The full extent of COVID-19 symptoms ranges from a benign, self-limiting respiratory condition to a merciless progressive pneumonia, multiple organ malfunction, and death (2–4). As per the World Health Organization (WHO), 753,823,259 cases including 6,814,976 deaths of COVID-19 were reported globally and in Bangladesh, there have been 2,037,578 confirmed cases of COVID-19 with 29,442 deaths, as of February 02, 2023 (5). To combat COVID-19 globally, several vaccines have acquired conditional approval (6). On January 27, 2021, COVID-19 vaccination began in Bangladesh with AstraZeneca (ChAdOx1-S/Covishield; manufactured by Serum Institute of India Pvt Ltd). To date, eight additional COVID-19 vaccines, i.e. Moderna/Spikevax (mRNA-1273), Gamaleya (Sputnik V), Pfizer-BioNTech/Comirnaty (BNT162b2), Sinopharm (BBIBP-CorV/Vero Cells), Johnson & Johnson/Janssen (Ad26.COV2.S), Oxford AstraZeneca: Vaxzevria, Sinovac (CoronaVac) and Novavax/COVOVAX (NVX-CoV2373) have received approval from the Government of Bangladesh (7). The Pfizer-BioNTech and Moderna vaccines use lipid nanoparticles to deliver spike-encoding mRNA. Adenovirus vector vaccine includes AstraZeneca, Gamaleya, and Johnson & Johnson/Janssen, while a protein subunit vaccine represents Novavax/COVOVAX. All these vaccines use the spike protein of the SARS-CoV-2 that first appeared in Wuhan, China, as the focal immunogen. To compare, the Sinopharm (BBIBP-CorV) and Sinovac (CoronaVac) are inactivated whole-virus vaccines that contain diverse viral proteins with possibilities of broadening immune protection beyond the spike-protein-specific immune response against the variants of concern (VOCs).

Currently 13 million Bangladeshis are engaged in various professions abroad. One of the key cornerstones of the Bangladeshi economy is the migrant labor force, which accounts for more than 12% of the total Gross Domestic Product (GDP) and 9% of all employment in Bangladesh (8–11). Almost all developed countries in the world implemented travel restrictions and border closures for migrant workers due to the coronavirus outbreak (12). Migrant workers have been caught between health and food crisis, the uncertainty of job retention, and have a continual desire to return to work for their livelihood (12). Restrictions on the entry of migrant workers has a significant negative impact on Singapore’s construction, marine, and process (CMP) sectors as well as employment generation, remittance earning and economic growth in Bangladesh. The leading associations of the CMP sectors began an industry-led pilot program in June 2021 to address the labor shortfall and aid in industry recovery (13). The pilot program relied on testing the workers using a COVID-19 testing regime over a 14-day period at specific in-house quarantine facilities in their home countries before their travel to Singapore in order to ensure a consistent intake of migrant workers in a safe and secure manner (13). During the pilot program, workers underwent rapid antigen tests, COVID-19 RT-PCR and serology tests to determine their current or past infection and antibody response after vaccination to COVID-19 (14, 15).

To measure antibodies to a range of SARS-CoV-2 antigens, such as spike protein (S) and nucleocapsid protein (N), several serological tests have been developed (16, 17). The S protein of SARS-CoV-2 contains a receptor-binding domain (RBD), which binds to angiotensin-converting enzyme 2 (ACE2) receptor located on the surface of the host cell, facilitating the entry of virus into the cell. Thus, the S protein is a key target for virus inactivation and assessment of immune response after vaccination (18). Associated with the viral genome, the nucleocapsid (N) protein is generated in enormous amounts in the early stages of infection. There is no cross-reactivity seen with N-specific antibodies even with closely related viruses (19). Both proteins are used as essential antigens in COVID-19 serology testing because of their strong immunogenicity (20).

The socioeconomic effects of COVID-19 on Bangladeshi migrant workers have been assessed in few studies (9, 21). Few other researchers have reported the immunological response to SARS-CoV-2 infection and the COVID-19 vaccination in Bangladeshi population (22–24). However, there is no report on seroconversion or post-vaccine COVID-19 antibody response in migrant workers. In the present study, we aimed to assess post-vaccination COVID-19 antibody response in Bangladeshi migrant workers to facilitate their migration to host countries. We also aimed to observe how vaccine types and previous SARS-CoV-2 infections influenced the antibody response. To achieve the objective, we evaluated SARS-CoV-2-S and SARS-CoV-2-N antibody responses in Singapore-outgoing Bangladeshi migrant workers.

## Methods

### Study design and population

**Figure 1:**
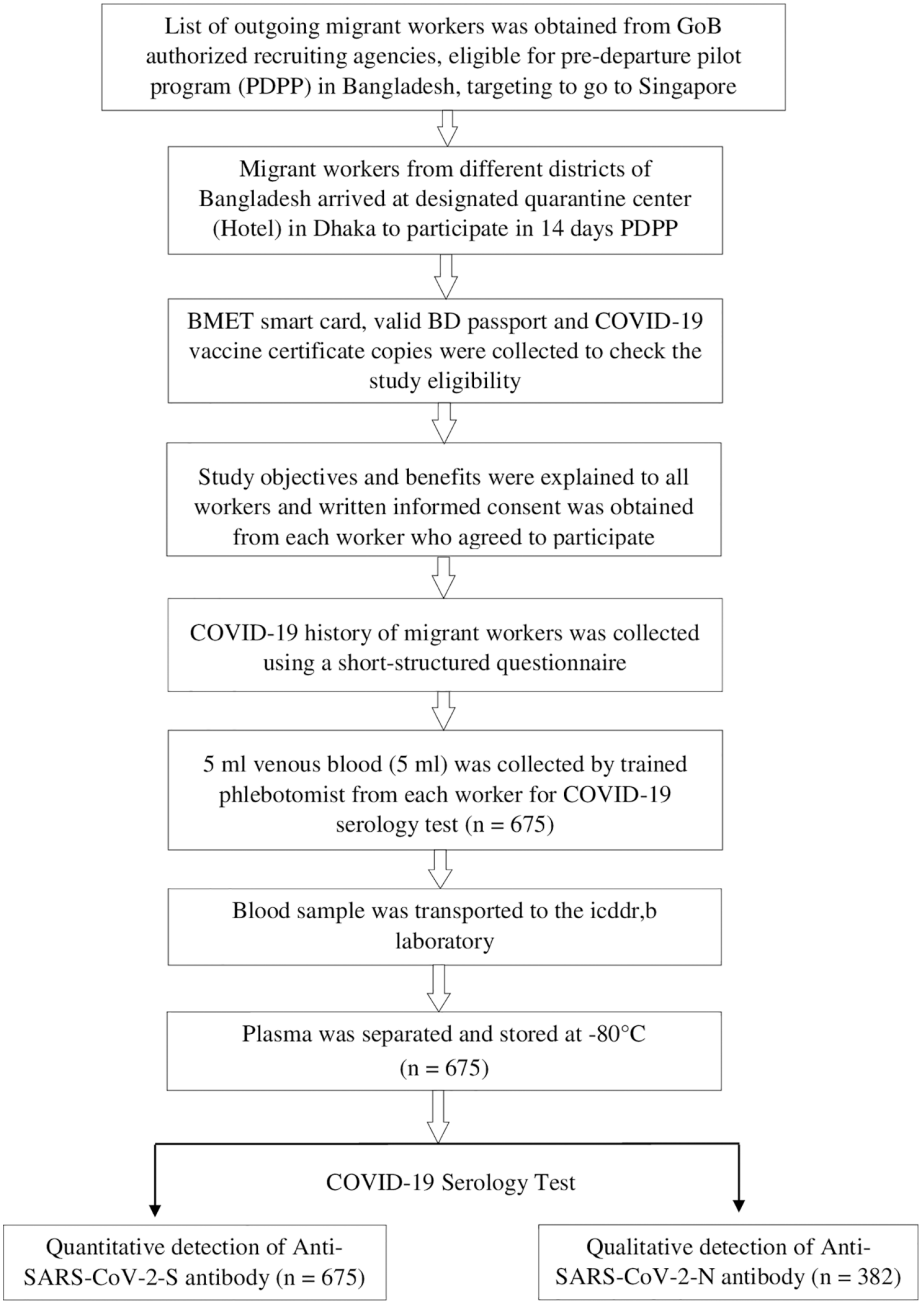
Flowchart showing enrollment, data and sample collection, and evaluation of COVID-19 post vaccine antibody response among the migrant workers, Bangladesh, 2021-22.

We conducted a cross-sectional study from December 2021 to February 2022 among the legal migrant workers of different districts of Bangladesh, who were eligible to participate in the pre-departure pilot program conducted by an international health service provider assigned by the Singapore Government (**Figure 1**). Migrant workers who met the following inclusion criteria were chosen for participation in the pilot program: holding a Bureau of Manpower Employment and Training (BMET) emigration clearance card, valid Bangladeshi passports, and a previous COVID-19 vaccine certificate. Those who failed to meet the inclusion criteria were excluded from the study.

### Study approval and ethical consideration

A written approval was obtained from the Ministry of Expatriates’ Welfare and Overseas Employment (MoEWOE), Bangladesh to conduct this study. The Institutional Review Board of International Centre for Diarrhoeal Disease Research, Bangladesh reviewed and approved the study protocol under the protocol # PR-21093. The ethical principles adhered to the present Helsinki Declaration as well as the statutory needs of the country. Prior to enrolment, each participant provided written informed consent.

### Enrolment of study participants and data collection

A Singapore outgoing migrant worker list was obtained from a Bangladesh Govt. authorized recruiting agency in Dhaka. The workers’ list contained their date of birth, passport number, home district, mobile number, quarantine center’s address, check-in date, and time. Migrant workers arrived at designated quarantine centers, which were located in several hotels in Dhaka, on the appointed date and time to participate in the host country’s required 14 days pilot program. Using a structured questionnaire, we collected information related to the date of vaccination for different doses, the type of COVID-19 vaccine received (from the COVID-19 vaccine card) and the self-reported history of SARS-CoV-2 infection of each worker.

### Blood sample collection

Single venous blood (~5 mL) was collected from participants (n=675) in anti-coagulant containing tubes (BD vacutainer^®^ Sodium heparin). All collected samples were transported in a portable cool box to the icddr,b laboratory within three hours of collection for further processing and laboratory analysis.

### Laboratory analysis

Plasma was separated from heparinized whole blood by centrifuging at 1900g at room temperature using a centrifuge (Eppendorf^®^ 5702R, Hamburg, Germany). After separation, plasma samples were tested for antibodies to SARS-CoV-2-S (n=675) and SARS-CoV-2-N (n=382) antigens by electrochemiluminescence immunoassay (ECLIA) using Elecsys Anti-SARS-CoV-2 S and Elecsys Anti-SARS-CoV-2 test kit (Roche Diagnostics, Indiana, USA). Both assays were carried out using the Roche Cobas-e601 immunoassay analyzer (Roche Diagnostics GmbH, Mannheim) following the manufacturer’s guidelines. In antibody detection, ECLIA can be compatible with the plaque reduction neutralization test (PRNT), microneutralization test (MNT), Pseudovirus Neutralization Assay (PNA) as revealed in the previous studies (24–26).

#### Anti−SARS−CoV−2-N antibody test

Elecsys Anti−SARS−CoV−2 is a qualitative immunoassay designed to detect antibodies (IgG and IgM) to the SARS−CoV−2 nucleocapsid (N) antigen in human serum/plasma. The software dictated the results automatically by comparing the electrochemiluminescence signal acquired from the reaction product of the sample with the signal of the previously calibrated cutoff value. The results were derived as sample/cutoff signal (COI) values and were qualitatively assessed as non-reactive (COI < 1.0; negative) or reactive (COI ≥ 1.0; positive). PreciControl Anti−SARS−CoV−2 method of Roche Diagnostics, USA was used for quality control.

#### Anti−SARS−CoV−2-S antibody test

Elecsys Anti−SARS−CoV−2 S is a quantitative immunoassay for the detection of antibodies to the receptor-binding domain (RBD) of the spike (S) antigen of SARS-CoV-2 in human serum or plasma. Anti−SARS−CoV−2 IgG, IgA and IgM antibodies in serum or plasma bind to specific recombinant antigens of SARS−CoV−2 S−RBD in a double-antigen sandwich assay format allowing quantitative determination of high-affinity antibodies through electrochemiluminescence technique. Results were obtained using a standard curve provided by the reagent barcode or e-barcode and a calibration curve that is instrument-specifically developed by two-point calibration. The analyte concentration of each sample was automatically computed by the analyzer in Units per milliliter (U/mL) and the numerical values were classified as “positive” (≥ 0.80 U/mL) and as “negative” (< 0.8 U/mL).

The WHO International Standard for anti-SARS-CoV-2 immunoglobulin (human), NIBSC code: 20/136, behaves identically to the internal Roche standard, with a correlation coefficient r = 0.9996 between Limit of Quantitation and 1000 BAU/mL (Binding Antibody Units (BAU)). Hence, the numeric results in U/mL of the Elecsys Anti SARS-CoV-2 S assay and BAU/mL are equivalent.

### Statistical analysis

Stata 14.2 (StataCorp LLC, College Station, Texas, USA) and R Studio version 1.4.1106 were used for statistical analysis and graph preparation. To determine whether the data was normal, the Kolmogorov-Smirnov test and histograms with normal curves were used. The antibody level was expressed as the median and interquartile range (IQR) and visualized using Boxplots with jitter, whilst categorical data were expressed as proportions/percentages. The Mann-Whitney U test and Kruskal Wallis test were used to compare median antibody levels between groups and time intervals since the last vaccination. We also used the Poisson regression model with robust standard error to evaluate the effects of multiple factors on antibody response to COVID-19 vaccination. The model was adjusted for co-variates such as age, vaccine doses, vaccine types, past SARS-CoV-2 infection, time since last vaccination. Scatterplots were constructed between age and antibody titers. The correlation coefficient of immune response with age, SARS-CoV-2 infection, number of vaccine doses received, different vaccine types, and anti-N antibody response was calculated using Spearman’s rank correlation. P-value < 0.05 represents statistical significance.

## Results

### Age, vaccination, and SARS-CoV-2 infection history of study participants

**Table 1** shows the sex, age category, COVID-19 vaccination status, SARS-CoV-2 infection history of the migrant workers, and the time interval between the last dose of COVID-19 vaccination and SARS-CoV-2 antibody testing. Migrant workers included in the study were all male. The mean age of the workers was 32 years, and most were between 18-40 years. More than 86% of the workers received double doses of vaccines, 11.1% received a single dose, and only 2.4% received a booster dose. About one-third of the migrant workers were vaccinated with Sinopharm/BBIBP-CorV (33%), followed by Pfizer/Comirnaty (28%), Moderna/Spikevax (26%), AstraZeneca/Covishield (13%), and mixed vaccine doses (0.60%). The median time interval between the last dose of COVID-19 vaccination and blood sample collection ranged from 32 days (for workers receiving a single dose of vaccine) to 109 days (for workers receiving double doses of vaccine). About 14% of the migrant workers reported that they were infected with SARS-CoV-2 in the last six months.

**Table 1:** Age, sex, vaccination, and SARS-CoV-2 infection history of the migrant workers.

Characteristics	All workers (n=675)
**Mean age (SD)**	**32 (7.44)**
Age category (year)	n (%)
18-40	588 (87.11)
41-51	87 (12.89)
Sex	
Male	675 (100)
Different COVID-19 vaccines received by the workers	
Sinopharm (BBIBP-CorV)	224 (33.19)
Pfizer (Comirnaty)	188 (27.85)
Moderna (Spikevax)	174 (25.78)
AstraZeneca (Covishield)	85 (12.59)
Mixed vaccine (S/M/P) ^*^	4 (0.59)
Number of vaccine doses received by the workers	
Single dose	75 (11.11)
Double dose	584 (86.52)
Booster dose	16 (2.37)
Past SARS-CoV-2 infection	
Yes	94 (13.93)
No	581 (86.07)
Frequency of reported past infection	
Once	94 (100)
Duration of reported past infection	Median (IQR)
Month	6 (16)
Time differences between last vaccination and blood collection for COVID-19 sero-survey (Day) collection for COVID-19 sero-survey (Day)	Median (IQR)
Overall	106 (101.50)
Single dose recipients	32 (208.50)
Double dose recipients	109 (92.30)
Booster recipients	90 (66.80)

*Spikevax plus Comirnaty and Comirnaty plus Covishield for double dose vaccine recipients; (Spikevax plus BBIBP-CorV and BBIBP-CorV) and (BBIBP-CorV plus BBIBP-CorV and Comirnaty) for booster dose vaccine recipients.

### Anti‐S seroconversion status based on age, previous infection with SARS-CoV-2 and number of doses

**Figure 2:**
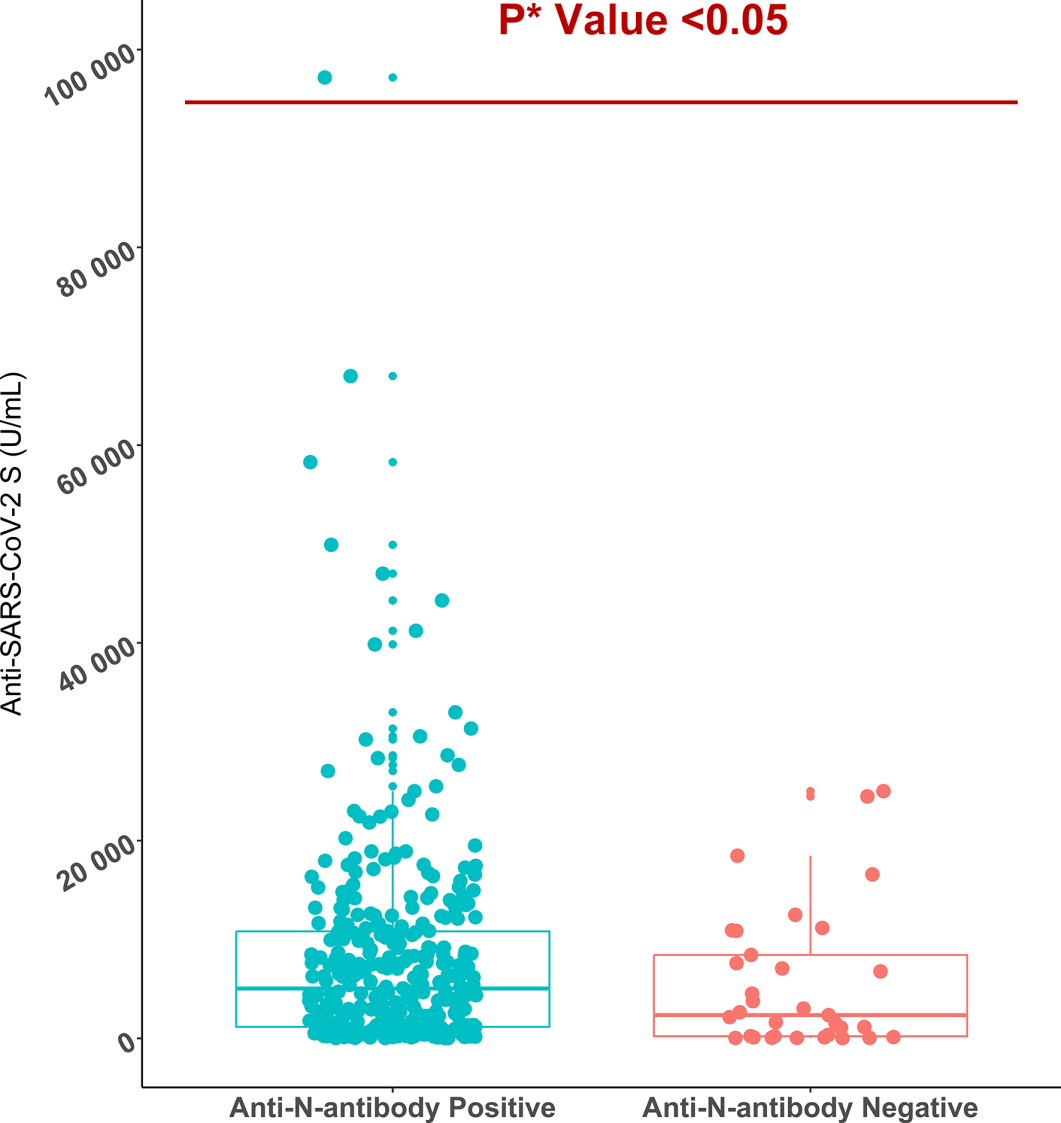
Anti-SARS-CoV-2 S antibody titers in anti-SARS-CoV-2 N antibody positive (n=349) and negative migrant workers (n=33). *P value is generated by Poisson regression model and the model was adjusted for age, vaccine types, and time since last vaccination.

**Figure 3:**
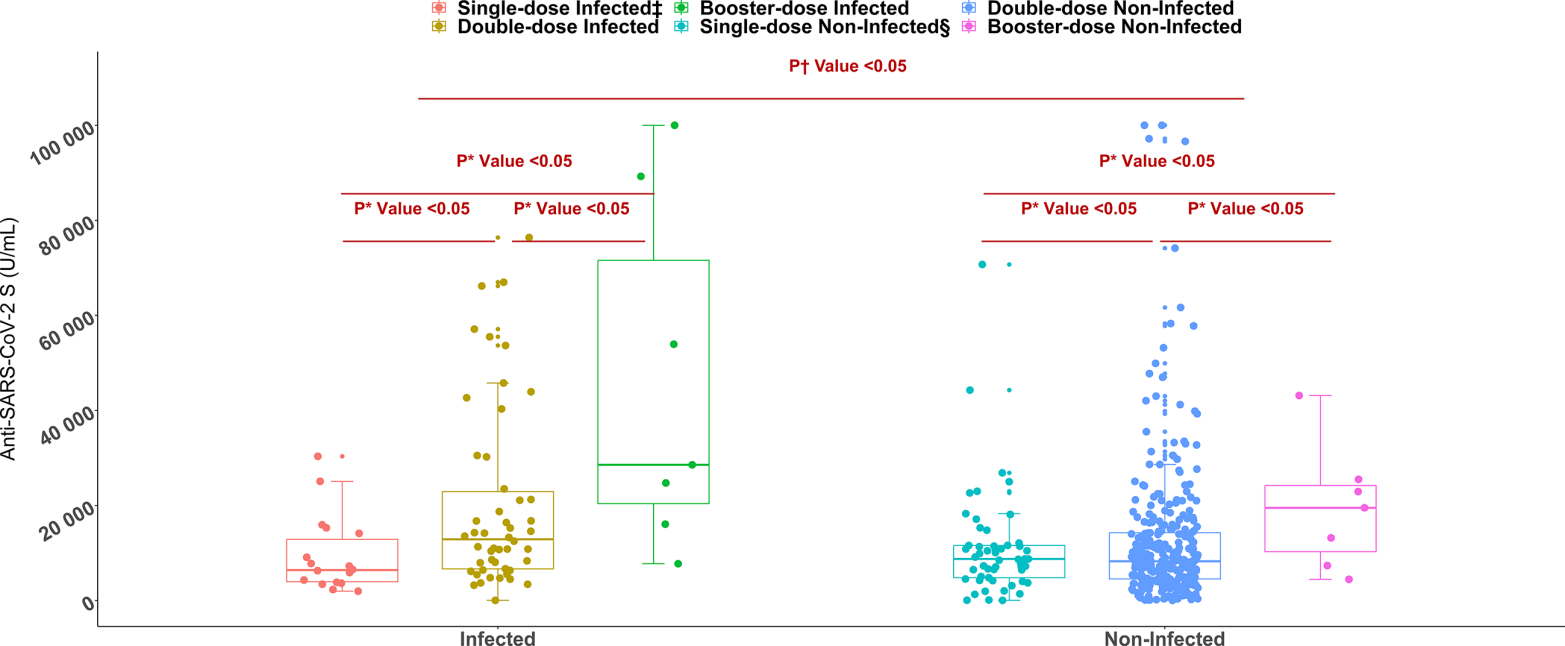
Anti-SARS-CoV-2 S antibody titers among single, double, and booster dose recipients of SARS-CoV-2 S antigen targeted vaccines (n= 447, Moderna/Spikevax, Pfizer/Comirnaty, AstraZeneca/Covishield). *P value is generated by Poisson regression model and the model was adjusted for age, vaccine types, and time since last vaccination. ^†^P value is generated by Mann Whitney U test. ^‡^Infected = workers infected with SARS-CoV-2, ^§^Non-Infected = workers who didn’t infect with SARS-CoV-2.

All workers were positive for S- antibodies and the average (median) titer of anti-S antibody was 6437 U/mL (IQR: 9713 U/mL, Range: 28.77 – 100000 U/mL). There was no discernible difference in S-antibody titers between the workers in the two age groups (18-40 years vs 41-51 years). Previous infection with SARS-CoV-2 had profound effect on vaccine-induced S-antibody titers. The S-antibody titer in SARS-CoV-2 infected workers was significantly higher than that in uninfected workers was (p < 0.001) (**Table 2**). Again, the S-antibody titer was significantly higher in the workers who were anti-N antibody positive (5193 U/mL) compared to the workers who were anti-N antibody negative 2357 U/mL (**Figure 2**). Multivariate analysis also showed significantly higher antibody response in SARS-CoV-2 infected (3347 U/mL) and anti-N antibody positive workers (2375 U/mL) compared to uninfected and anti-N antibody negative workers (**Table 3**). Among the SARS-CoV-2 infected workers (self-reported; 14%), the highest antibody concentration (28563 U/mL) was detected in booster dose (3^rd^ dose) recipients followed by workers receiving double doses (10416 U/mL) and single dose (6410 U/mL) of the vaccine. Among the uninfected workers (86%), the highest anti-S titer (13327 U/mL) was noted after receiving booster dose, followed by 8499 U/mL after single dose and 5273 U/mL after double dose (**Table 2**). Among the infected workers who were vaccinated with SARS-CoV-2 S antigen-targeted vaccines, anti-S antibody concentration were significantly higher in participants receiving booster dose compared to participants receiving single and double doses. Again, double dose recipients showed significantly higher antibody response than single dose recipients. Similar response was observed for booster dose recipients in uninfected workers, however, double dose recipients showed lower response than single dose recipients (**Figure 3**). Significantly higher antibody titers in booster dose and double dose recipients compared to single dose recipients were also evident in multivariate Poisson regression analysis (**Table 3**).

**Table 2:** COVID-19 post vaccine Anti−SARS−CoV−2−S antibody response among the migrant workers, Bangladesh, 2021-22.

Variables	Anti−SARS−CoV−2−S antibody (N=675) [U/mL]			
Median (IQR)	Infected (n=94)		Non-infected (n=581)	
8849 (13997)		6013 (9525)	
P value^*^	<0.001			
Age (year)	Median (IQR)	P value^†^	Median (IQR)	P value^†^
18-40	8849 (16750)	0.755	6209 (9671)	0.775
41-51	9296 (7702)		4850 (9315)	
Vaccine doses				
Single dose	6410 (10304)	0.006	8499 (7270)	0.001
Double dose	10416 (12042)		5273 (9492)	
Booster dose	28563 (73192)		13327 (15568)	

^*^P value is generated between SARS-CoV-2 infected and non-infected recipients by Mann Whitney U test.

^†^P value is generated by Kruskal Wallis test.

**Table 3:** Multivariate analysis for Anti-S antibody response to COVID-19 vaccines among the migrant workers, Bangladesh, 2021-22.

Variables	Coefficient	P-value*	95% CI
Vaccine Doses			
Single dose	Ref		
Double dose	1181.428	<0.001	(1153.132, 1209.723)
Booster dose	17839.58	<0.001	(17741.03, 17938.14)
Different COVID-19 Vaccines			
AstraZeneca (Covishield)	Ref		
Moderna (Spikevax)	5679.379	<0.001	(5648.231, 5710.527)
Pfizer (Comirnaty)	3557	<0.001	(3526.803, 3587.197)
Sinopharm (BBIBP-CorV)	-6234.395	<0.001	(-6261.249, -6207.542)
Past SARS-CoV-2 infection			
No	Ref		
Yes	3347.866	<0.001	(3321.804, 3373.928)
Anti-N Antibody			
Negative	Ref		
Positive	2375.98	<0.001	(2348.739, 2403.221)

^*^P value is generated by Poisson regression.

### Anti-S seroconversion status with different vaccine types

**Figure 4:**
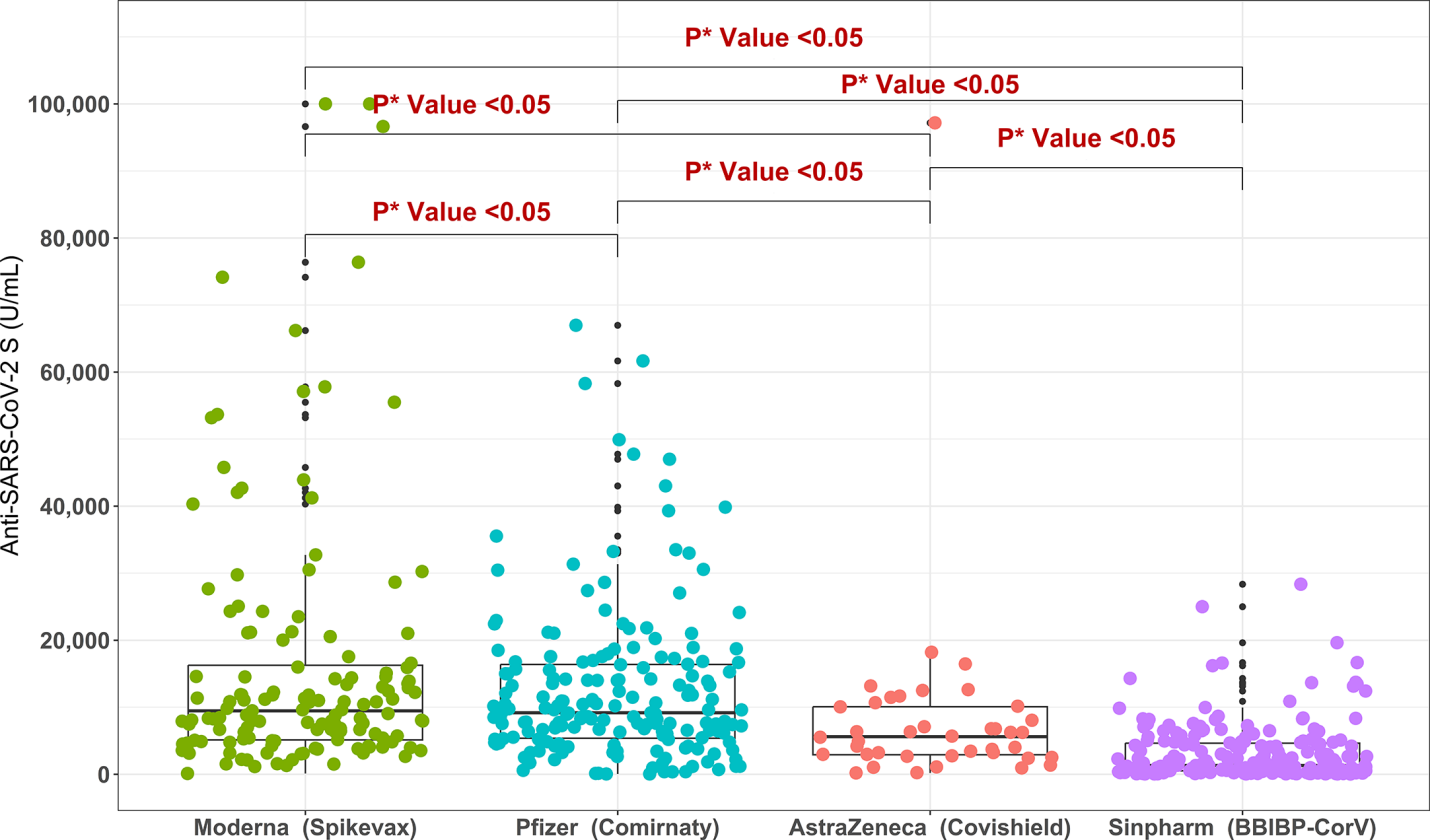
Anti-SARS-CoV-2 S antibody titers in migrant workers receiving double doses of different COVID-19 vaccine types. *P value is generated by Poisson regression model and the model was adjusted for age, past SARS-CoV-2 infection and time since last vaccination.

For the migrant workers who received double doses of COVID-19 vaccine, the highest anti-S antibody titer was found for Moderna/Spikevax (9459 U/mL) and Pfizer/Comirnaty (9181 U/mL) vaccines, followed by AstraZeneca/Covishield (5601 U/mL) and Sinopharm/BBIBP-CorV (1308 U/mL) vaccines. m-RNA base vaccines Moderna/Spikevax and Pfizer-BioNTech/Comirnaty elicited significantly higher anti-S antibody titers compared to vector-based Astrazeneca/Covishield and Sinopharm/BBIBP-CorV vaccines. Between Astrazeneca/Covishield and Sinopharm/BBIBP-CorV vaccinated participants, anti-S antibody concentration was significantly higher for the AstraZeneca/Covishield (**Figure 4**). Similarly, in multivariate analysis, migrant workers receiving AstraZeneca/Covishield vaccine showed significantly lower response compared to Moderna/Spikevax and Pfizer/Comirnaty vaccine recipients, but the response was significantly higher compared to Sinopharm/BBIBP-CorV vaccine recipients (**Table 3**). Among the workers who received booster doses, the highest anti-S antibody level (28563 U/mL) was found in Moderna/Spikevax vaccine recipients followed by 25498 U/mL in Pfizer/Comirnaty, 10023 U/mL in mixed vaccines, and 7551 U/mL in AstraZeneca/Covishield vaccine recipients (**Supplementary Table 1**).

### Anti‐S seropositivity at different time intervals since last vaccination

**Figure 5:**
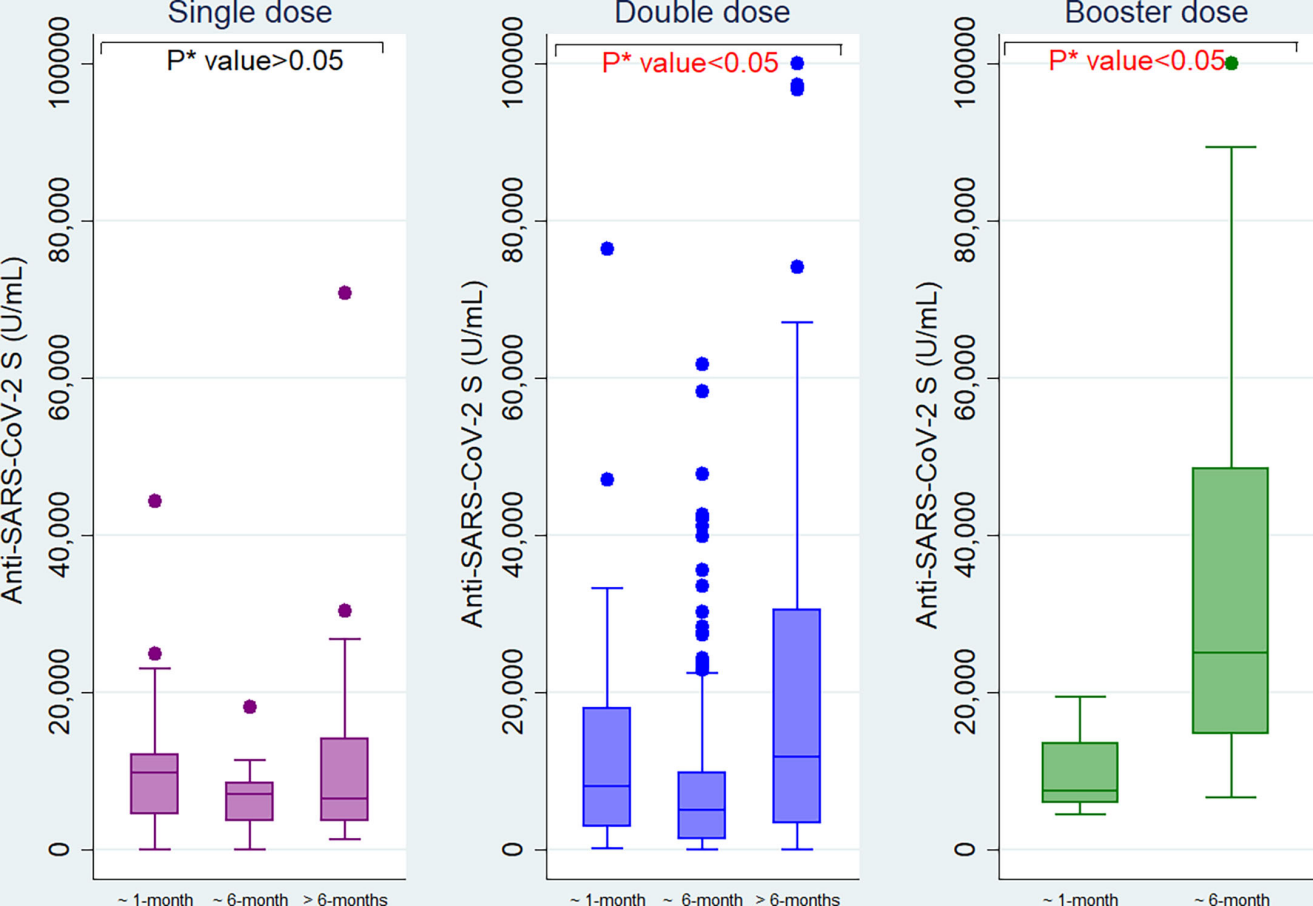
Anti−SARS−CoV−2 S antibody titers among the migrant workers at different time intervals since last vaccination. *P value is generated by Kruskal Wallis test.

Among migrant workers receiving single dose of vaccine, anti-S antibody titer declined over time since last vaccination (from 9878 U/mL within one month to 7076 U/mL at six months intervals and 6500 U/mL after greater than six months). For the workers who were given double doses, the antibody titer was 8184 U/mL within one month of vaccination, significantly reduced to 5094 U/mL at six months interval and increased again at later time point (11861 U/mL). Among booster vaccine recipients, the antibody titer was increased from 7551 U/mL within one month of vaccination to 25120 U/mL at the six months interval (**Figure 5**).

### Correlation of anti-S antibody with age, vaccine dose, anti-N antibody response, vaccine types, and SARS-CoV-2 infection

**Figure 6:**
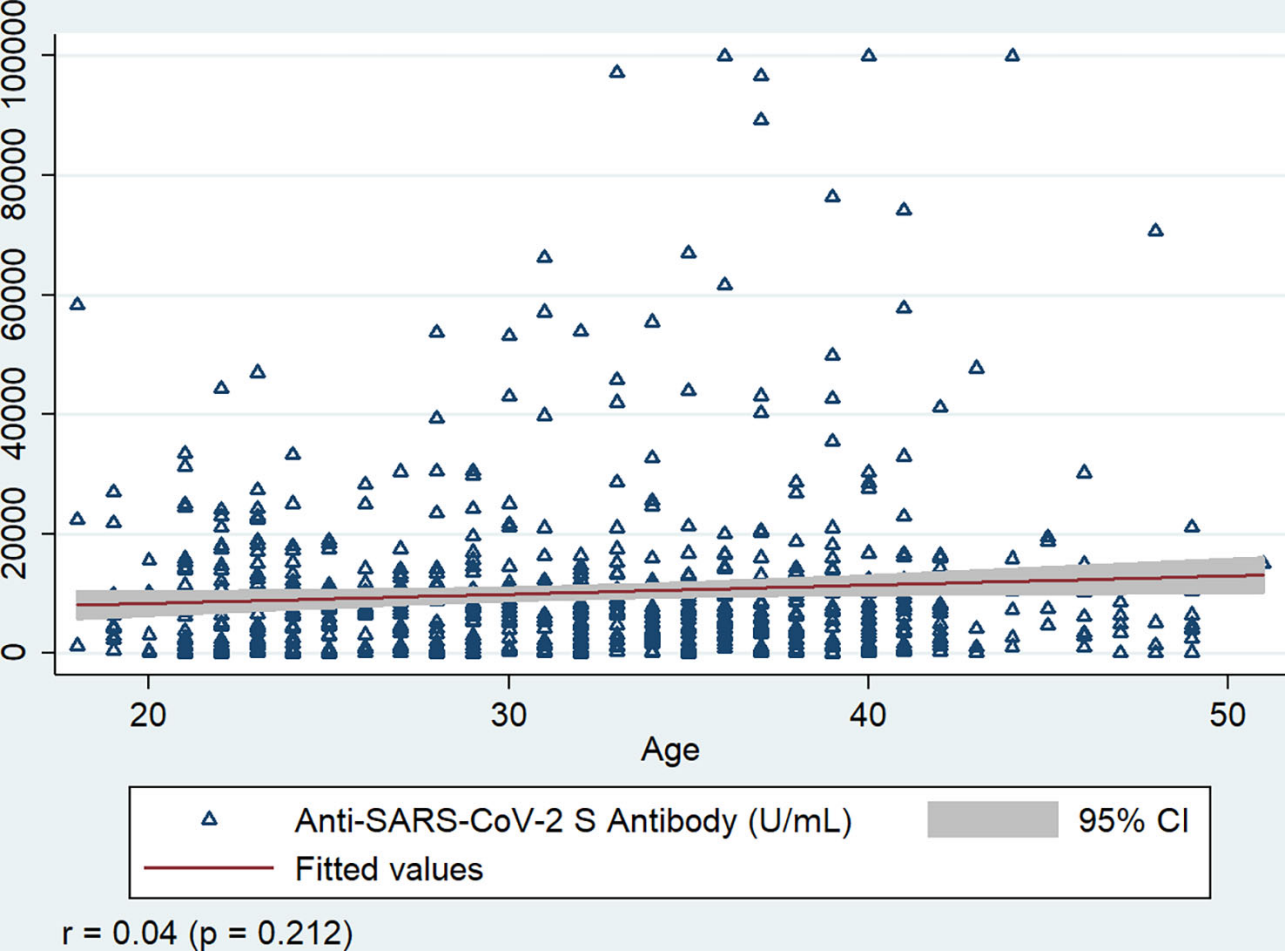
Correlation of Anti-SARS-CoV-2 S antibody with age of the workers, Bangladesh, 2021-22. r = Spearman’s rho, P value is generated by Spearman’s rank correlation.

Anti-S titers were highly correlated with different types of vaccines received (r = - 0.441, p<0.001), and moderately correlated with past SARS-CoV-2 infection (r = 0.183, p<0.001) and anti-N antibody response of the workers (r = 0.108, p = 0.034). Anti-S antibodies did not show a significant correlation with age (r = 0.048, p = 0.212), and the number of vaccine doses received by the workers (r = 0.006, p = 0.875) (**Figure 6**).

## Discussion

To our knowledge, this is the first scientific study assessing antibody response to different COVID-19 vaccines among Bangladeshi migrant workers. This study demonstrated higher anti-S antibody titers in workers receiving Moderna/Spikevax and Pfizer/Comirnaty vaccines compared to AstraZeneca/Covishield and Sinopharm/BBIBP-CorV vaccine recipients. In addition, higher anti-S antibody titers were observed in migrant workers who have received booster doses of vaccine as well as among those previously infected with SARS-CoV-2. Furthermore, antibody titers were found to decline after six months since the last vaccination.

Our result suggested that mRNA vaccines (Moderna/Spikevax and Pfizer/Comirnaty) elicited significantly higher anti-S antibody titers in Bangladeshi migrant workers compared to the viral vector-based vaccine (AstraZeneca/Covishield) and inactivated vaccine (Sinopharm/BBIBP-CorV). This result is consistent with findings from several immune response studies on COVID-19 vaccines in human populations (27–31). In a recently conducted community-based study in Bangladesh, mRNA vaccine recipients showed higher antibody response than adenovector and killed whole-virus vaccine recipients (24). The S protein of SARS-CoV-2 is used as the immunogen in the Pfizer/Comirnaty, Moderna/Spikevax, and AstraZeneca/Covishield vaccines to generate anti-S antibodies that block the S protein’s binding to host cell (32–34). The AstraZeneca/Covishield vaccine uses S-protein coding DNA inserted in a chimpanzee adenovirus vector (35). Conversely, the Moderna/Spikevax and Pfizer/Comirnaty vaccines employ codon-optimized mRNA sequences supplied to the host cell *via* lipid nanoparticles (LNPs) that is directly translated into full-length S protein (36). Higher antibody response of mRNA vaccines compared to adenovector vaccines may be explained by quick mRNA transfer into the host cell by LNP, effective S protein synthesis, and stabilizing alterations blocking the structural change in the S protein (36–38). In the case of the whole cell Sinopharm/BBIBP-CorV vaccine, the concurrent decoupling of S1 and synthesis of the post-fusion S by inactivation and purification methods may lessen the antibody response (36). mRNA vaccines may provide superior protection in comparison to inactivated and vector-based vaccines through higher antibody response (35, 39).

Antibody titers were found to be higher in the workers receiving booster doses compared to double and single doses (p<0.001) vaccine recipient, irrespective of the previous history of SARS-CoV-2 infection. Immunization with two doses of the vaccine also mounted a higher antibody response than a single dose in the workers with the exception of non-infected vaccine recipients. These findings were similar to previously studied vaccine dose-based antibody responses among healthcare workers (HCWs) and healthy individuals in Italy and India (40, 41). Ward H et al., also cited greater antibody response following vaccination in double-dose vaccine recipients than single-dose recipients among general populations in England (42). A study conducted among healthy individuals in the USA reported that all participants experienced significant elevation of measured antibodies following the second vaccination dosage, even those who had a weak or negative reaction to the first dose/shot of vaccine (43). A statistically significant relationship of antibody response was also found between single and double dose vaccine recipients in the Bangladeshi population (44). The lower anti-S antibody of non-infected double dose vaccine recipients than single dose recipients in our study may have resulted from longer time interval between last vaccination and sample collection (**Table 1**).

Age is one of the most crucial factors affecting the antibody response. Age-related declines in T-cell-derived antibody production and B-lymphocyte formation may result in a diminished antibody response to infectious pathogens or vaccinations (45). The post-vaccination antibody response was found to be inversely proportional to age in numerous studies conducted following immunizations against pneumococcus, tetanus, hepatitis A, hepatitis B, influenza, tick-borne encephalitis (TBE), and SARS-CoV-2 (16, 46–48). However, age and antibody production did not have a significant relationship in our study. The non-significant relationship between age and antibody in our study is most likely due to narrow age range (18-51 years). In our study, we did not have elderly participants (> 60 years) because the study participants were working-age population departing Singapore. Observation of our study blends well with studies carried out in Turkey and Egypt (49, 50).

We found the highest anti-S antibody in workers who reported previous SARS-CoV-2 infection compared to the workers who did not report SARS-CoV-2 infection. A strong correlation between past SARS-CoV-2 infection and higher anti-S antibodies was also seen. Recently, in five major divisions of Bangladesh, persons with previous SARS-CoV-2 infection were shown to have higher post-vaccine immunological responses compared to non-infected individuals (24). In another study in the Bangladeshi population, after immunization, those with a record of SARS-CoV-2 infection had six times higher antibody titers than those without a history of infection (51). Healthcare workers in Italy also showed a 10 to 100-fold rise in anti-S antibody and neutralizing antibody titers who had already contracted SARS-CoV-2 (52). Collectively, these studies including ours strongly suggest the role of immunological memory after a natural infection or vaccination in generating rapid and high response to subsequent exposure. Conversely, another Italian investigation found no correlation between the level of anti-S antibodies and a prior SARS-CoV-2 infection (53).

Our study demonstrated that antibody titers were significantly reduced at a six-month interval since the last COVID-19 vaccination among the workers. Consistent with this finding, community-based COVID-19 sero-epidemiological studies among healthy blood donors and healthcare workers in Hong Kong and South Korea revealed reduction of antibody levels over six months following Comirnaty and CoronaVac vaccination (54, 55). It was also reported that after vaccination with double doses of Comirnaty vaccine, antibody levels declined substantially at 6 months intervals since the last vaccination (56, 57). Several other studies have reported a waning of antibody response to different vaccines with time and highlighted the importance of providing booster doses (58–60). In our study, the rise in antibody titers after six months post-vaccination in double and booster dose recipients is likely due to a breakthrough infection of SARS-CoV-2 at that time.

## Study limitations and way forward

Our study has several limitations. The major limitation of our study was that data and samples were collected at a single-time point, as the migrant workers participated in the study just before departure to the host country. Consequently, we were unable to assess the participants’ long-term antibody response and safety profile. It is known that antibody level can vary based on sex (61, 62); our limitation was that we had only male participants as only male migrant workers went through the Singapore outgoing pilot program during the study period. Another limitation was the small sample size in some categories, e.g., the single and booster dose recipients of different COVID-19 vaccines; and infected people within different vaccine types. Moreover, the infection data of SARS-CoV-2 could not be confirmed by PCR.

## Conclusion

A robust antibody response was observed among the migrant workers who reported past SARS-CoV-2 infection, were vaccinated with mRNA vaccines, and completed booster doses. However, antibody level significantly decreased over six months since the last vaccination, which warrants provision of further booster doses among the migrant workers, specially before departure. Regular monitoring of serological response is necessary for such programs to confirm the safety profile of the migrant workers. Moreover, this sero-monitoring initiative will help formulate appropriate policy regarding the migrant workers health and infection control during the ongoing and future pandemics by respective governments (source and host countries), local and international migrant-focused organizations, and Non-Governmental Organizations (NGOs).

## Data availability statement

The data will be shared based on the demand or upon a reasonable request to the corresponding author from any reader.

## Ethics statement

The studies involving human participants were reviewed and approved by International Centre for Diarrhoeal Disease Research, Bangladesh Institutional Review Board. The patients/participants provided their written informed consent to participate in this study.

## Author contributions

MH: Conceptualization, Methodology, Validation, Investigation, Data curation, Software, Formal analysis, Visualization, Writing – original draft, Review & editing. PS: Conceptualization, Methodology, Validation, Formal analysis, Visualization, Writing – original draft, Review & editing. RR: Conceptualization, Methodology, Validation, Writing – Review & editing, Supervision. MZR: Conceptualization, Methodology, Formal analysis, Data curation, Writing – Review & editing. RH: Conceptualization, Methodology, Formal analysis, Data curation, Writing – Review & editing. CS: Conceptualization, Methodology, Data curation, Writing – Review & editing, Visualization. MR: Conceptualization, Methodology, Formal analysis, Data curation, Writing – review & editing, Supervision. NA: Conceptualization, Methodology, Validation, Formal analysis, Data curation, Writing – original draft, Review & editing, Visualization, Supervision. All authors contributed to the article and approved the submitted version.
