# Supplementary material for: Antibody response to different COVID-19 vaccines among the migrant workers of Bangladesh
Source: Front Immunol. 2023 Mar 9;14:1128330. doi: 10.3389/fimmu.2023.1128330 (PMC10034009; doi:10.3389/fimmu.2023.1128330)
Supplement: Supplementary file 1 [file Table_1.docx]

Supplementary Material

**Antibody response to different COVID-19 vaccines among the migrant workers of Bangladesh**

**Md. Imam Hossain^1^, Protim Sarker^1^, Rubhana Raqib^1^, Md Ziaur Rahman^1^, Rezaul Hasan^1^, Chloe K. Svezia^2^, Mahbubur Rahman^1^, Nuhu Amin^1,3, *^**

^1^Infectious Diseases Division, International Centre for Diarrhoeal Disease Research, Dhaka, Bangladesh

^2^Rollins School of Public Health, Emory University, Atlanta, USA

^3^Institute for Sustainable Futures, University of Technology Sydney, 235 Jones St., Ultimo, NSW 2007, Australia

*** Correspondence:**Nuhu Amin
nuhu.amin@icddrb.org

Supplementary Table 1: COVID-19 post vaccine Anti‑SARS‑CoV‑2‑S antibody response with different vaccine brands and frequency of vaccine doses among the migrant workers, Bangladesh, 2021-22.

| **Doses and different vaccines** | | **Anti‑SARS‑CoV‑2‑S antibody (N=91) [U/mL]** | |
| --- | --- | --- | --- |
|  |  | **n** | **Median (IQR)** |
| ***Single dose recipients*** | Pfizer | 2 | 34641(19282) |
|  | Moderna | 28 | 6391 (9053) |
|  | AstraZeneca | 41 | 8765 (4963) |
|  | Sinopharm | 4 | 532 (267) |
| ***Booster dose recipients*** | Pfizer | 3 | 25498 (31005) |
|  | Moderna | 7 | 28563 (73192) |
|  | AstraZeneca | 4 | 7551 (7720) |
|  | Sinopharm | 0 | 0 |
|  | Mixed (S+M/P) ^*^ | 2 | 10023 (6607) |

^*^(Moderna plus Sinopharm and Sinopharm) and (Sinopharm plus Sinopharm and Pfizer).
